# Holographic and e-Beam Image Recording in Ge_5_As_37_S_58_–Se Nanomultilayer Structures

**DOI:** 10.1186/s11671-016-1235-x

**Published:** 2016-01-27

**Authors:** A. Stronski, E. Achimova, O. Paiuk, A. Meshalkin, V. Abashkin, O. Lytvyn, S. Sergeev, A. Prisacar, G. Triduh

**Affiliations:** V. Lashkaryov Institute of Semiconductor Physics, National Academy of Science of Ukraine, 41 Nauki ave., Kiev, 03039 Ukraine; Institute of Applied Physics, Academy of Sciences of Moldova, 5 Academiei str., Chisinau, 2028 Moldova

**Keywords:** Multilayer nanostructures, Holographic recording, e-beam recording, Diffraction gratings, Chalcogenide glasses

## Abstract

Processes of e-beam and holographic recording of surface relief structures using Ge_5_As_37_S_58_–Se multilayer nanostructures as registering media were studied in this paper. Optical properties of Ge_5_As_37_S_58_, Se layers, and Ge_5_As_37_S_58_–Se multilayer nanostructures were investigated. Spectral dependencies of refractive index were analyzed within the frames of single oscillator model. Values of optical band gaps for Ge_5_As_37_S_58_, Se layers, and Ge_5_As_37_S_58_–Se multilayer nanostructures were obtained from Tauc dependencies. Using e-beam and holographic recording, diffraction gratings were fabricated in Ge_5_As_37_S_58_–Se multilayer nanostructures. Images of Ukraine and Moldova state emblems were obtained by e-beam recording. Image size consisted of 512 × 512 pixels (size of 1 pixel was ~2 μm). Ge_5_As_37_S_58_–Se multilayer nanostructures are perspective for the direct recording of holographic diffraction gratings and other optical elements.

## Background

Thin films based on chalcogenide glasses have evolved as light-sensitive materials for high-density recording media application due to their optical and structural properties. The light sensitivity effect of thin chalcogenide films was discovered 50 years ago [1]. Chalcogenide glasses and films are also sensitive to the electron or ion beams, X-rays [2–5], and perhaps photo-stimulated or stimulated by electron or ion beams; X-rays’ change of their properties is the most interesting phenomena exhibited by these materials.

In this work, the experimental results showing the surface relief formation in Ge_5_As_37_S_58_–Se nanomultilayer structures under e-beam or holographic exposure are presented.

Selective etching after exposure enables to obtain surface reliefs and to use media such as high-resolution inorganic resists [2–7]. Using of thin layers of chalcogenide glasses as high-resolution media and selective etching after exposure enables fabrication of high-quality holographic diffraction gratings and other optical elements [6–11].

Multilayer nanostructures on the base of chalcogenide glasses and the possibility to use them as registering media were proposed in [12]. Such media do not require the step of selective etching for the formation of the surface relief [12–16]. Surface relief in such media is formed directly during exposure process. Absence of the selective etching step is the advantage of such media because often USED etchants are toxic, and during selective etching process, it is necessary to control many parameters (temperature, concentration of etchant, etc.). Thus, the development of one-step method for the fabrication of surface reliefs is considered perspective for the fabrication of planar optical elements.

Chalcogenide glasses of Ge–As–S composition are characterized by high values of refractive index, and their nonlinear optical properties are two orders higher than characteristic of quartz glasses [17, 18]. Earlier, we demonstrated the possibility to use Ge_5_As_37_S_58_–Se multilayer nanostructures for the fabrication of surface reliefs [15]. In present work, results of direct recording (without the selective etching step) of holographic diffraction grattings and images by e-beam exposure using Ge_5_As_37_S_58_–Se multilayer nanostructures as recording media are presented.

## Methods

**Fig. 1:**
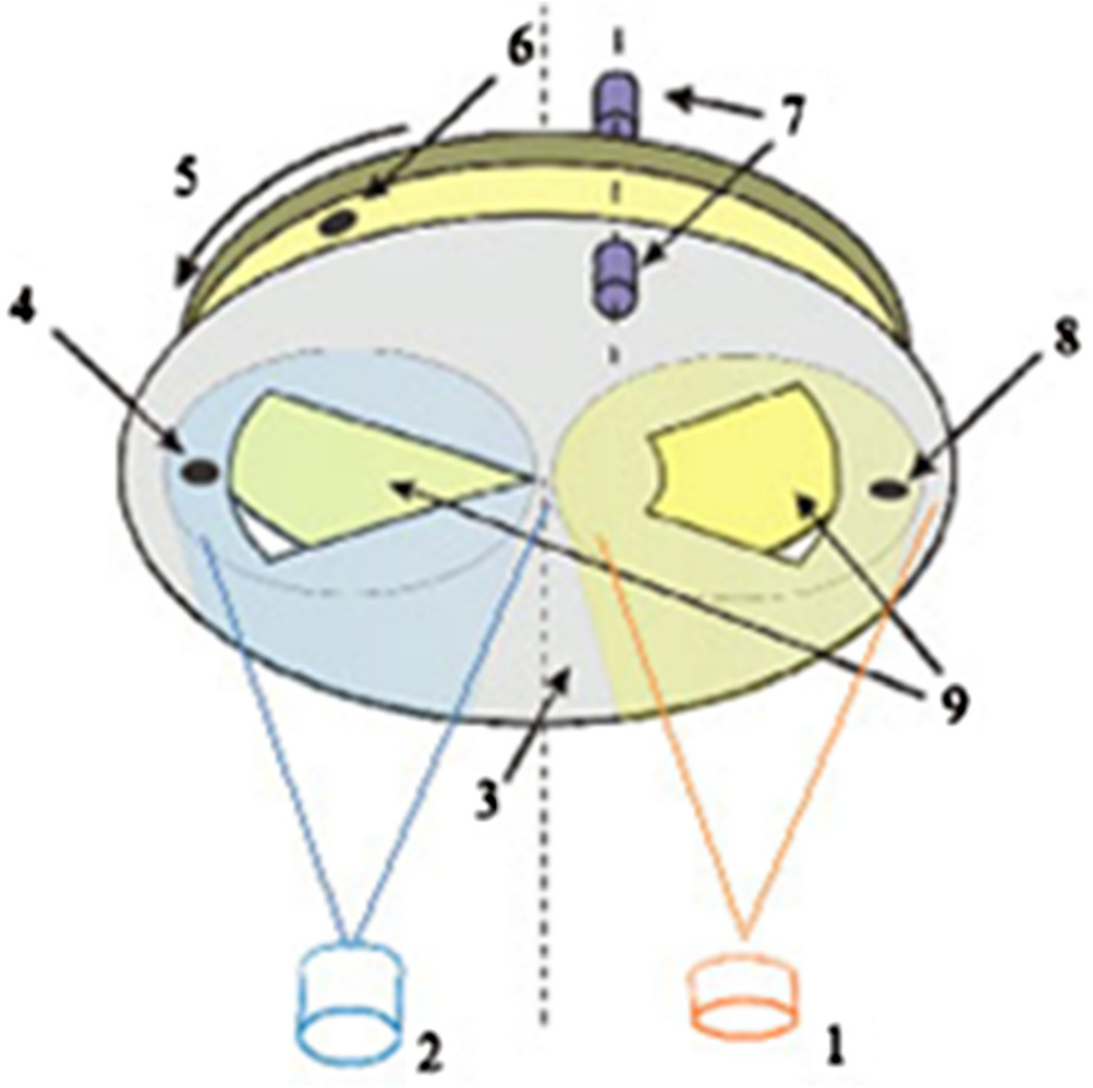
Scheme of device for fabrication of multilayer nanocomposites on the base of chalcogenide glasses. *1* Evaporate of Ge-As-S chalcogenide glass, *2* evaporator of Se, *3* stationary mask, *4* and *8* quartz thickness sensors fixed on mask, *5* rotating samples holder, *6* quartz thickness sensor fixed on the rotating samples holder, *7* optical fibers of spectrophotometer, *9* windows in mask

Bulk glasses of Ge_5_As_37_S_58_ were fabricated by common melt quenching method [15, 17, 18]. Amorphous Ge_5_As_37_S_58_–Se nanomultilayers were prepared by computer-driven cyclic thermal vacuum deposition from two isolated boats with Ge_5_As_37_S_58_ and Se on constantly rotated glass substrate with deposited ITO layer at room temperature in one vacuum deposition cycle (Fig. 1).

**Fig. 2:**
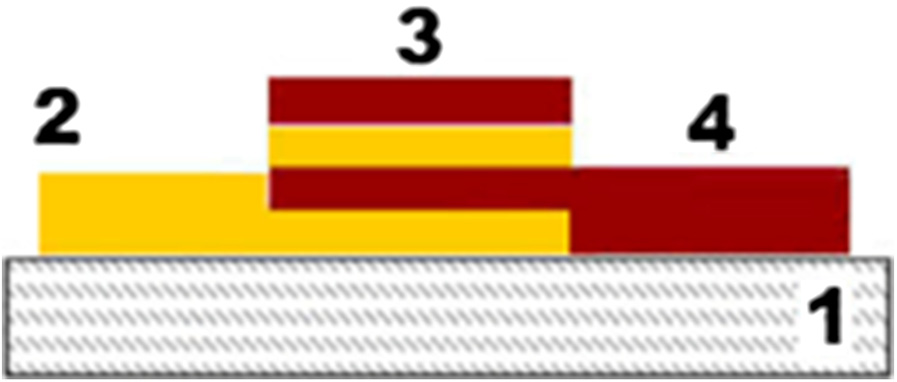
Scheme of the sample structure. *1* Glass substrate, *2* Ge_5_As_37_S_58_ layer, *3* Ge_5_As_37_S_58_–Se multilayer nanostructure, *4* Se layer

The control of the film thickness was carried out in situ during the thermal evaporation by interference thickness sensor at *λ* = 0.95 μm. Scheme of transverse cross section of the sample is shown in Fig. 2, where 1 is the glass substrate, 2 Ge_5_As_37_S_58_ layer deposited nanolayer by nanolayer, 3 Ge_5_As_37_S_58_–Se multilayer nanostructure, and 4 Se layer deposited nanolayer by nanolayer.

Overlapping part of the sample consists of alternating Se and Ge_5_As_37_S_58_ nanolayers; two wide rings are overlapping in the central part of the substrate forming the Ge_5_As_37_S_58_–Se multilayer nanostructure. Consequently, external and inner rings of the layers are Ge_5_As_37_S_58_ and Se layers. Ge_5_As_37_S_58_ and Se were deposited through mask windows. The substrate with deposited multilayer structure and Ge_5_As_37_S_58_ and Se layers was used for the composition control and also for AFM thickness measurement and estimation of modulation period *N* (common thickness of one Ge_5_As_37_S_58_ nanolayer and one Se nanolayer) of multilayer nanostructure. Overlapping part of the samples (see Fig. 2.) contains alternating nanolayers of Ge_5_As_37_S_58_ with thickness of 16 nm and Se nanolayers with thickness of 14 nm. The total number of nanolayers was 200, modulation period *N* ~ 30 nm. In order to prevent crystallization of Se layers which are structurally unstable under heating and/or exposure by light, e-beams, etc., heating of layers was minimized by substrate rotation and lowered evaporator temperature.

**Fig. 3:**
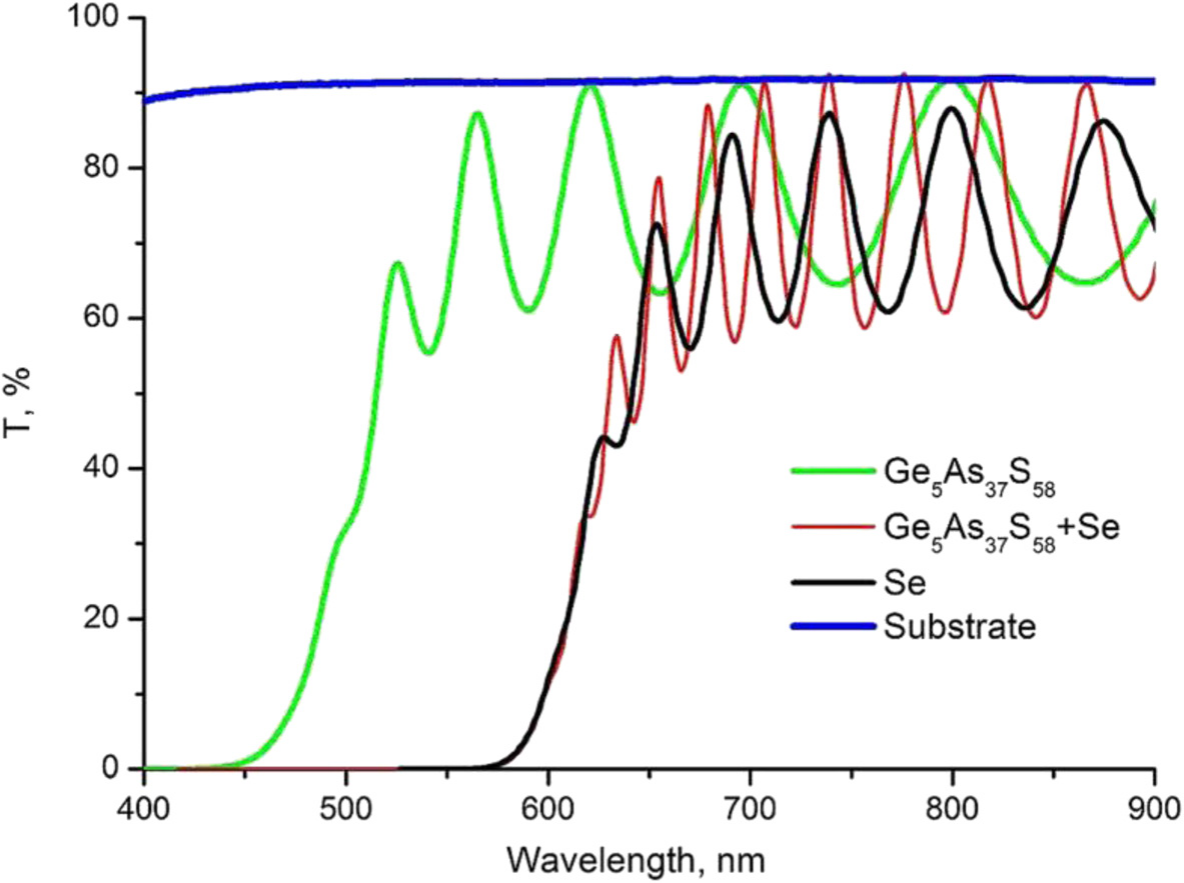
Optical transmission spectra of Ge_5_As_37_S_58_, Se layers, and Ge_5_As_37_S_58_–Se multilayer nanostructure

Optical properties of fabricated films and structures were investigated with the use of optical transmission spectra measured at normal light incidence. Transmission spectra (Fig. 3) were measured in the 450–900-nm range with the use of Specord M40 spectrophotometer for obtaining thickness, optical band gap, and spectral dependencies of refractive index of Ge_5_As_37_S_58_, Se layers, and Ge_5_As_37_S_58_–Se multilayer nanostructure.

**Fig. 4:**
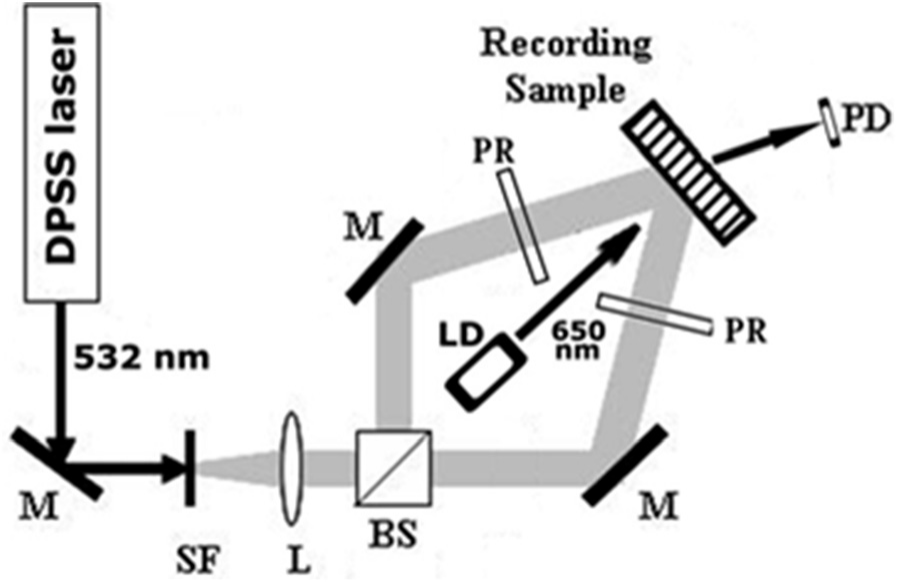
Optical arrangement for holographic grating recording with real-time measurement of diffraction efficiency by photodetector. CW DPSS laser (*λ* = 532 nm, power =100 mW). *M* Mirror, *SF* spatial filter, *L* collimating lens, *BS* beam splitter, *LD* laser diode (*λ* = 650 nm) for monitoring recording process, *PR* polarization retarder, *PD* photodetector

Recording of holographic gratings was carried out with the use of DPSS laser radiation on 532 nm wavelength; diffraction efficiency of the diffraction gratings was controlled during recording process using 650 nm wavelength. Recording scheme is shown in Fig. 4.

Diffraction gratings of Ukrainian and Moldavian state emblems were recorded by e-beam exposure using scanning electron microscope Tesla BS 300 with programmable exposure control unit. The accelerating voltage was 25 kV, and the size of the electron spot at this voltage was about 300 nm. Size of images consisted of 512 × 512 pixels. Morphology and surface relief of the obtained images were studied by AFM microscopy. Distance between pixels consisted of 3 μm. Size of pixels was about 2 μm and profile depth ~300 nm.

## Results and Discussion

The refractive index, *n*, and the film thickness were calculated using Swanepoel’s method [19]. Algorithms and computer program for the calculation of envelope curves and refractive index were similar to those presented in [20–23]. The method allows the calculation of *n* when both the refractive index of the substrate and the position of the interference extrema are known. In the present study, the refractive index, *s*, of the substrate was determined independently at various wavelengths by measuring the transmittance, *T*_s_, of the substrate alone (see curve 4 Fig. 3) and using the following equation [24]:

1 $$ s=1/{T}_{\mathrm{s}}+{\left({T}_{\mathrm{s}}^2-1\right)}^{1/2}. $$

For chalcogenide semiconductors, the optical absorption coefficient, *α*, changes rapidly for photon energies comparable to that of the band gap, *E*_g_, giving rise to an absorption edge with three regions—for the largest electron energies, in the region of the edge itself (10 < *α* < 10^4^ cm^−1^), and at the lowest photon energies [25]. The first one is for the highest values of the absorption coefficient (*α* ≥ 10^4^ cm^−1^) which corresponds to transitions between extended states in both valence and conduction bands where the power law of Tauc [26] is valid.

2 $$ \alpha (hv)=\frac{B{\left(hv-{E}_{\mathrm{g}}\right)}^2}{hv} $$

*B* is the slope of the Tauc edge which reflects some disorder of the samples. Usually, this constant depends on the width of the localized states in the band gap, a fact explained with the homopolar bonds’ presence in the chalcogenide glasses. Thus, Tauc plots of (*αhν*)^1/2^ versus (*hν*) should be linear and extrapolate to values of the optical gap, *E*_g_.

The dispersion of the refractive index was analyzed using the Wemple–DiDomenico (WDD) model [27, 28], which is based on the single oscillator formula:

3 $$ {n}^2-1={E}_{\mathrm{d}}{E}_0/\left({E}_0^2-{E}^2\right), $$

where (*hv*) is the photon energy, *E*_0_ is the oscillator energy, and *E*_d_ is the oscillator strength or dispersion energy. The parameter *E*_d_ which is a measure of the intensity of the inter-band optical transition does not depend significantly on the band gap. By plotting (*n*^2^ − 1)^−1^ against (*hν*)^2^ and fitting a straight line to the points, *E*_d_ and *E*_0_ can be directly determined from the slope, (*E*_d_*E*_0_)^−1^, and the intercept *E*_0_/*E*_d_ on the vertical axis. The extrapolation for (*hv*)→0 also gives frequency-independent refractive index or the so-called static refractive index, *n*_0_.

The dispersion energy is related to other physical parameters of material through the empirical formula:

4 $$ {E}_{\mathrm{d}}=\beta {N}_{\mathrm{c}}{Z}_{\mathrm{a}}{N}_{\mathrm{e}}, $$

where *N*_c_ is the effective coordination number of the cation nearest neighbor to the anion, *Z*_a_ is the formal chemical valency of the anion, *N*_e_ is the total number of valence electrons per anion, and *β* is a constant (with a value depending on whether the solid is covalent or ionic, *β* = 0.26 ± 0.03 and *β* = 0.37 ± 0.04 eV, respectively). As found by Wemple and DiDomeniko [27], the oscillator energy *E*_0_ is closely related to the optical band gap energy *E*_g_.

As can be seen from Fig. 3, absorption edge of Ge_5_As_37_S_58_–Se multilayer nanostructures practically coincides with the absorption edge of Se (see also Table 1). It is necessary to note (see Fig. 3) high optical quality of Ge_5_As_37_S_58_ layers and Ge_5_As_37_S_58_–Se multilayer nanostructures (film transmission coincides with the substrate transmission in sites of interference maxima) and the presence of scattering in Se layer (transmission values of interference maxima for Se layers are smaller than values of substrate transmission).

**Table 1:** Parameters of single oscillator model and values of optical band gap for Ge_5_As_37_S_58_, Se films, and Ge_5_As_37_S_58_–Se multilayer nanostructures

Layer composition	*n*(0)	*E*_d_, еВ	*E*_0_, еВ	*E*_g_, еВ
Se	2.28	19.09	4.53	1.91
Ge_5_As_37_S_58_	2.24	18.29	4.57	2.27
Ge_5_As_37_S_58_–Se	2.37	17.67	3.84	1.92

The thicknesses of constituent Ge_5_As_37_S_58_ and Se nanolayers were 16 and 14 nm, respectively, and are sufficiently smaller than the light wavelength. Also, transmission curve of Ge_5_As_37_S_58_–Se multilayer nanostructure is a typical interference curve for films with high optical quality and uniform thickness (see Fig. 3). Thus, in the analysis of optical transmission spectra of nanomultilayer structure, it was possible to use the “effective optical medium” model: the layers with small optical band gap *E*_g_ value determine the optical absorption at the average absorption edge *E*_g_, and the “barrier” layers with larger *E*_g_ are transparent.

Obtained spectral dependencies of refractive index of Ge_5_As_37_S_58_, Se layers, and Ge_5_As_37_S_58_–Se multilayer nanostructures were analyzed within the frames of single oscillator model. Parameters of the model (dispersion energy, position of the effective oscillator) were obtained.

Parameters of single oscillator model dispersion energy and effective oscillator position and also values of optical band gap obtained with the use of Tauc dependence *αhν =* const(*hν − E*_g_)^2^, where *hν* is the light quantum energy, *α* absorption coefficient, and static refractive index, *n*_0_, are presented in Table 1.

It is necessary to note that values of optical band gaps (see Table 1) of Se layers and Ge_5_As_37_S_58_–Se multilayer nanostructure are close. The Wemple–DiDomenico model in the range of the low-frequency optical dielectric response of glasses can be used as a tool to probe the building blocks conforming a glass and allows quantitative analysis in combination with Raman spectroscopy [29].

Mechanism of recording in chalcogenide multilayer nanostructures is connected with stimulated by light (ion, electron beams) interdiffusion processes in nanolayers [30]. The proposed model for low-intensity recording radiation enabled to calculate evolution of the recorded reliefs during holographic recording of gratings. Further improvement of this model was done in [31], where heating of multilayer nanostructure during recording process by high-intensity light and respective non-linearities of the recording processes were taken into account. Also, it is noted that the thickness change as a result of interdiffusion processes in alternating nanolayers can reach ~5 % values and more. In mechanisms of surface relief formation, it is also necessary to take into account peculiarities of possible surface relief formation in constituent layers of multilayer structure (Se and chalcogenide layer of other composition) [32]. Here, it is necessary to note that mechanisms, processes of mass transfer during surface relief formation, and kinetics of holographic gratings recording in chalcogenide layers and in nanomultilayer structures on the base of chalcogenide glasses are polarization sensitive [32–35].

**Fig. 5:**
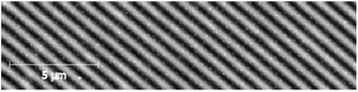
AFM image of surface of holographic diffraction grating with spatial frequency 1000 mm^−1^, obtained on the base of Ge_5_As_37_S_58_–Se multilayer nanostructure

In Fig. 5, AFM image of surface of holographic diffraction grating with spatial frequency of 1000 mm^−1^, obtained on the base of Ge_5_As_37_S_58_–Se multilayer nanostructure, is shown.

Under exposure by ion- or e-beams of thin layers of chalcogenide, vitreous semiconductors structural transformations in films are observed [2–5, 36–38]. Shifts of absorption edge or surface deformation under e-beam exposure were observed. With the use of consequent selective etching thin layers of chalcogenide, glasses can be applied in high-resolution lithography processes. It was noted in [36–38] that the effects of stimulated by e-beam thickness increase in separate components of multilayer nanostructure (for example, Se and As_2_S_3_) are not additive in respect to thickness increase in the same conditions for Se–As_2_S_3_ multilayer nanostructure. The sum of the thickness increase in separate layers consists of only 30 % from the total measured thickness increase in Se–As_2_S_3_ multilayer nanostructures. In multilayer nanostructures, it is necessary to take into account also the presence of other processes, for example, formation of As–S–Se. It is supposed that stimulated by light or e-beams processes activate the mentioned processes in such media.

**Fig. 6:**
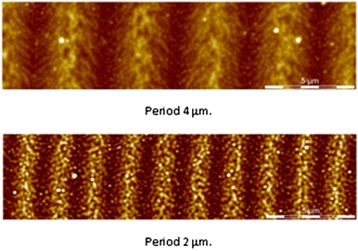
Diffraction gratings obtained with the use of e-beam recording

In Fig. 6, AFM images of diffraction gratings recorded by e-beam exposure with 4 and 2 μm periods are presented. Diffraction efficiency of the gratings was ~1 %. Further investigations are necessary for the optimization of multilayer nanostructure parameters and conditions of grating recording.

**Fig. 7:**
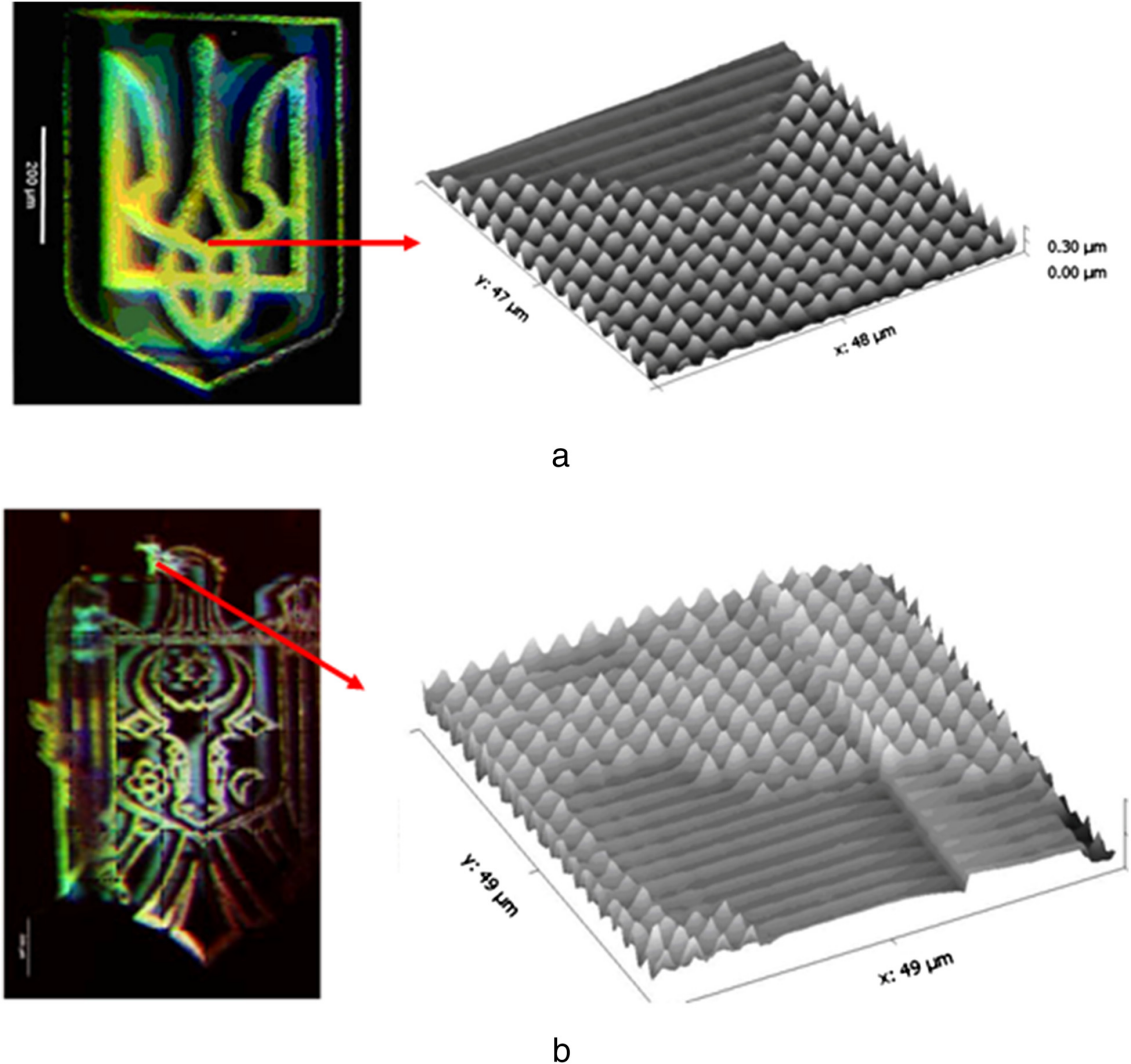
Results of e-beam pixel recording of Ukraine (**a**) and Moldova (**b**) state emblems with the use of Ge_5_As_37_S_58_–Se multilayer nanostructure. AFM images of the recorded emblem fragments are shown on the right sides of figures

In Fig. 7a, the results of e-beam pixel recording of the Ukraine state emblem with the use of Ge_5_As_37_S_58_–Se multilayer nanostructure are shown. Image sizes of the Ukraine state emblem consisted of 512 × 512 pixels (pixel size ~2 μm). AFM image of the recorded emblem fragment is shown on the right side of Fig. 7a. It is necessary to note that at the given recording conditions, pixel height is up to 200–300 nm. Figure 7b shows the results of e-beam pixel recording of the Moldova state emblem with the use of Ge_5_As_37_S_58_–Se multilayer nanostructure. AFM image of the recorded emblem fragment is shown on the right side of Fig. 7b.

## Conclusions

Obtained results show that with the use of Ge_5_As_37_S_58_–Se multilayer nanostructures, it is possible to realize direct recording of holographic optical elements (diffraction gratings) and also direct recording by e-beam exposure of diffraction gratings and other surface relief structures.
